# SOST gene suppression stimulates osteocyte Wnt/β-catenin signaling to prevent bone resorption and attenuates particle-induced osteolysis

**DOI:** 10.1007/s00109-023-02319-2

**Published:** 2023-05-01

**Authors:** Zixue Jiao, Hao Chai, Shendong Wang, Chunguang Sun, Qun Huang, Wei Xu

**Affiliations:** 1 Department of Orthopedics, The Second Affiliated Hospital of Soochow University, Suzhou, 215004 Jiangsu China; 2 Department of Orthopedics, Taiyuan Central Hospital of Shanxi Medical University, Taiyuan, 030009 Shanxi China; 3 Department of Orthopedics, Funing People’s Hospital, Yancheng, 224400 Jiangsu China; 4 Department of Orthopedics, Zhangjiagang City First People’s Hospital, Zhangjiagang, 215699 Jiangsu China

**Keywords:** Periprosthetic osteolysis, SOST, Wnt/β-catenin, Osteoclast, Bone resorption

## Abstract

**Abstract:**

The most common cause for prosthetic revision surgery is wear particle-induced periprosthetic osteolysis, which leads to aseptic loosening of the prosthesis. Both SOST gene and its synthetic protein, sclerostin, are hallmarks of osteocytes. According to our previous findings, blocking SOST induces bone formation and protects against bone loss and deformation caused by titanium (Ti) particles by activating the Wnt/β-catenin cascade. Although SOST has been shown to influence osteoblasts, its ability to control wear-particle-induced osteolysis via targeting osteoclasts remains unclear. Mice were subjected to development of a cranial osteolysis model. Micro CT, HE staining, and TRAP staining were performed to evaluate bone loss in the mouse model. Bone marrow-derived monocyte-macrophages (BMMs) made from the C57BL/6 mice were exposed to the medium of MLO-Y4 (co-cultured with Ti particles) to transform them into osteoclasts. Bioinformatics methods were used to predict and validate the interaction among SOST, Wnt/β-catenin, RANKL/OPG, TNF-α, and IL-6. Local bone density and bone volume improved after SOST inhibition, both the number of lysis pores and the rate of skull erosion decreased. Histological research showed that β-catenin and OPG expression were markedly increased after SOST inhibition, whereas TRAP and RANKL levels were markedly decreased. In-vitro, Ti particle treatment elevated the expression of sclerostin, suppressed the expression of β-catenin, and increased the RANKL/OPG ratio in the MLO-Y4 cell line. TNF-α and IL-6 also elevated after treatment with Ti particles. The expression levels of NFATc1, CTSK, and TRAP in osteoclasts were significantly increased, and the number of positive cells for TRAP staining was increased. Additionally, the volume of bone resorption increased at the same time. In contrast, when SOST expression was inhibited in the MLO-Y4 cell line, these effects produced by Ti particles were reversed. All the results strongly show that SOST inhibition triggered the osteocyte Wnt/β-catenin signaling cascade and prevented wear particle-induced osteoclastogenesis, which might reduce periprosthetic osteolysis.

**Key messages:**

- SOST is a molecular regulator in maintaining bone homeostasis.
- SOST plays in regulating bone homeostasis through the Wnt/β-catenin signaling pathway.
- SOST gene suppression stimulates osteocyte Wnt/β-catenin signaling to prevent bone resorption and attenuates particle-induced osteolysis.

**Supplementary Information:**

The online version contains supplementary material available at 10.1007/s00109-023-02319-2.

## Introduction

Artificial joint replacement has been widely employed in medical care for a range of end-stage joint disorders, femoral head necrosis, and trauma, which has a considerable pain-relieving impact and improves patients’ joint functioning and quality of life [1]. A complication of arthroplasty known as the aseptic loosening of the prosthesis has become more prevalent; there has been a rise in the use of prosthetic joints as the elderly population has grown in recent years [2]. Even though the mechanical source of aseptic loosening remains to be elucidated, periprosthetic osteolysis is caused by wear particles which are considered to be the significant runner for this process [2–4]. Some studies showed that wear particles of titanium (Ti), polymethylmethacrylate, polyethylene, and cobalt-chromium produced by artificial joints stimulated the RANKL/OPG/RANK system, directly stimulating osteoclast production and led to increased osteolysis [2, 5]. Wear particles can also activate inflammatory mediators such as TNF-α, IL-6, MMP-2, and others around the implant that are released by osteoclasts, macrophages, monocytes, fibroblasts, and osteoclasts. These mediators also help osteoclasts differentiate and prevent osteoblasts from maturing [4, 6–8]. The exact cause of periprosthetic osteolysis is yet unknown. Both the development and progression of periprosthetic osteolysis remain tough to prevent and control.

SOST gene and its protein product, i.e., sclerostin, a characteristic biomarker of osteocytes. SOST/sclerostin can inhibit Wnt signaling [9] and has a bidirectional regulatory role in bone remodeling [10, 11]. Sclerostin stimulates osteocyte secretion of RANKL and activates osteoclasts while inhibiting osteoblast differentiation and inducing osteoblast apoptosis. Some research have showed that SOST/sclerostin is involved in sclerosis, fracture repair, osteoporosis, and periprosthetic osteolysis [12, 13]. After SOST/sclerostin blockade, bone volume increases, and endophyte fixation improves in osteoporosis [14]. Our previous studies shown that blocking the SOST gene increases bone formation and compensates bone loss caused by Ti particles by activating the Wnt/β-catenin signaling pathway [15, 16]. It is known that SOST regulates osteoclasts by modulation of RANKL /OPG expression by osteocytes [17]. Though, still, it is undefined whether this mechanism is operative in wear-particle induced osteolysis.

The objective of the current study was to further explore SOST/sclerostin effects in the periprosthetic osteolysis process and to investigate whether inhibition of the SOST gene could inhibit osteoclastogenesis and hence alleviate wear particles induced-osteolysis by in-vitro and in-vivo studies.

## Materials and methods

### Ti particle preparation

Pure titanium (Ti) particles were obtained from Johnson Matthey (catalog #00681, Ward Hill). Manufacturer claims 90% of the Ti particles were less than 10.0 mm in diameter, with the average being 5.34 mm. In the earlier studies, the efficacy of these particles was demonstrated [15].

The particles were developed following the reported procedure of Chen et al. Furthermore, a QCL-1000 kit was employed to make sure that no endotoxin was present (BioWhittaker) [8]. Endotoxin-free Ti particles (100 mg/mL) were prepared and stored in PBS at 4 °C. For experiments on cells and animals, the Ti particle suspension was diluted to the desired concentration.

### SOST adeno-associated virus vector for in vivo experiments and development of a cranial osteolysis model

The approval for animal utility in this study was provided by the Second Affiliated Hospital of Soochow University’s ethics committee. Herein, 40 C57BL/6 female mice aged 8 weeks and weighing 20 to 25 g were selected. Four groups of mice (i.e., (a) Sham group, (b) Ti (Ti) group, (c) SOST-RNAi (SOST-L) group, and (d) SOST-RNAi + Ti (SOST-L + Ti) group) were randomly assigned to each group. Adeno-associated virus vector (50 μL) was subcutaneously injected in the center of the skull of each mouse. The viral vectors injected into the SOST-L and SOST-L + Ti groups carried 10^12^ replicas of the siRNA SOST gene. One week later, mice were anesthetized with 50 mg/kg pentobarbital (1%) intraperitoneally, and a 10-mm sagittal incision was made in the skull’s center. The sham and SOST-L groups received an equal volume of PBS injection, while the mice in the Ti group and SOST-L + Ti group received 50 μL of Ti particles in PBS suspension (100 mg/mL) under the periosteum of the sagittal suture of the skull. After 2 weeks, the skull samples of the mice were taken for radiological, histological, and immunohistochemical examinations.

### Micro-CT Analysis

The cranial specimens were examined via micro-CT (Scanoco) at 10 μm/layer after being preserved in 4% paraformaldehyde (*n* = 5 per group). The X-ray settings were 70 kV and 114 μA. The following data was obtained using micro-CT on a circular region of interest (ROI) with a 6-mm diameter in the middle of each skull: bone volume (BV), bone mineral density (BMD), bone volume/tissue volume (BV/TV), and the number of perforations.

### Histological and immunohistochemical analysis

Skull samples (*n* = 5 per group) were decalcified in 10% ethylenediaminetetraacetic acid (EDTA, Biosharp) for a month after being soaked in formalin for 2 days. The specimens were then trimmed, mainly to preserve the Ti particle-covered parietal and anterior bones of the mice models. After dehydration and paraffin embedding, the cranial specimens were proceeded for hematoxylin and eosin (H&E) staining and cut into 5-μm sections. Images of the H&E staining results were obtained with the skull midline suture located in its center at 20 × magnification. The surface area of bone tissue (BS mm^2^) and eroded bone surface area (ES mm^2^) were calculated using Image Pro-Plus 6.0 software.

Immunohistochemical staining technique was occurred to detect the appearance of the following proteins: sclerostin (Abcam, ab86465, 1:200 dilution), β-catenin (Proteintech, ab66379, 1:200 dilution), OPG (Abcam, ab183910, 1:500 dilution), and RANKL (Abcam, ab45039, 1:200 dilution). Furthermore, osteocytes were labeled with sclerostin, OPG, and RANKL, while osteoclasts were labeled with TRAP staining (Cosmo Bio Co. Ltd). First, skull specimens were dewaxed, gradient hydrated and antigen extracted. Primary antibodies were overnight incubated at 4 °C. Afterward, the tissue slices were first rinsed and incubated with a buffer containing secondary antibody for 35 min at room temperature. Each tissue sample was photographed about the median sagittal suture of the skull under a 20 × and 40 × microscope, respectively. Cells that stained brown were immunoreactive positive cells and positively stained cells were counted in three sections from every group (*n* = 5) by two independent observers under a 20 × microscope.

### Overexpression and silencing of SOST

The osteocytes MLO-Y4 cell lines were taken from the Chinese Academy of Sciences (CAS) cell bank and seeded in α-MEM containing 10% FBS (Gibco). Next, this culture mixture was incubated at 37 °C with a continuous supply of 5% CO_2_. Short-hairpin shRNA lentiviral particles taken for the Sigma-Aldrich Chemical Co. were used according to the provided protocol of the manufacturer to silence a portion of MLO-Y4 osteocytes. Briefly, following a GenBank search for the SOST gene sequence, the SOST shRNA interference sequence was designed using Invitrogen’s RNA interference and synthesized manually by Sangon Biotech. MLO-Y4 osteocytes were transfected via lentivirus units carrying disordered or SOST-specific shRNAs. Using Western blotting, the expression of the sclerostin protein was measured separately to see how well shRNA worked. The most efficient SOST-shRNA and SOST overexpressed sequences in MLO-Y4 osteocytes were screened and the detail sequences were in supplement Table 1. MLO-Y4 cells that expressed different levels of SOST were used in the next in vitro experiments.

### Cell culture and treatments

After 24 h after seeding, MLO-Y4 cells were treated with Ti particles at doses of 0, 0.1, and 1.0 mg/ml (control). The medium was replaced by adding fresh medium every 3 days. The day the Ti particles were introduced to the cells is known as Day 0. The MLO-Y4 osteocytes were then divided into four groups and inoculated in 6-well Corning plates: the control group, the Ti group, the SOST-L + Ti group, and the SOST-H group. For 48 h, Ti particles at 1.0 mg/ml were added to MLO-Y4 cells in the Ti and SOST-L + Ti groups. All groups had their initial culture medium for osteoclasts collected, centrifuged to remove any Ti particles or other impurities suspended therein, and then replaced every 2 days with fresh medium.

Primary bone marrow-derived monocyte macrophage (BMM) extraction then induction of differentiation had BMMs remained extracted from the bone marrow of tibias and femurs of 4-week-old female mice C57BL/6. The cells were seeded in α-MEM containing 10% FBS and were incubated overnight at 37 °C with a continuous supply of 5% CO_2_. Next, the supernatant was then centrifuged to obtain suspension cells, and these cell suspensions were resuspended in 10% FBS containing α-MEM and M-CSF solution (30 ng/ml) (R&D systems). After 3 days, the adherent cells in the above culture dishes were collected, transferred to 6-well plates at a density of 4 × 10^5^/well, and resuspended in α-MEM containing 50 ng/ml of RANKL (R&D systems), 30 ng/ml of M-CSF, and 10% FBS in α-MEM to stimulate their differentiation to osteoclasts. Cells after culturing for 3 days with TRAP staining and a nuclear number ≥ 3 were considered mature osteoclasts. Additionally, the characteristic markers of osteoclasts were identified (Supplement 1B-C).

In the indirect cell co-culture model, BMMs were induced to develop into osteoclasts using the method described above. Differently, when BMMs were transferred to 6-well plates, the medium that was collected from each group of MLO-Y4 osteocytes cultured, as described above, was added, with the addition of RANKL (50 ng/ml) and M-CSF (30 ng/ml). After the development of multinucleated cells, subsequent experiments were conducted.

### Immunofluorescence staining

After being rinsed three times in PBS, MLO-Y4 osteocytes were fixed in 4% paraformaldehyde for 10 min, permeabilized with 0.1% Triton X-100 for 5 min, and incubated in 0.1% PBS-Tween (PBST) containing 5% bovine serum albumin (BSA) for 1 h. Secondary, OPG (1:500 dilution) and RANKL (1:500 dilution) primary antibodies were incubated with MLO-Y4 osteocytes at 4 °C overnight. The cells were washed thricely using PBS, followed by exposure to goat anti-rat (Alexa Fluor® 594, Abcam, ab15016, 1:500 dilution), goat anti-mouse (Alexa Fluor® 647, Abcam, ab150115, 1:500 dilution), and goat anti-rabbit (Alexa Fluor® 488, Abcam, ab150077, 1:500 dilution) secondary antibodies for an hour in the absence of light. Cell nuclei were stained for 5 min with DAPI (1:1000 dilution) after being thricely washed with PBST.

BMMs were inoculated into confocal culture dishes and were induced to differentiate osteoclasts, fix, and break membranes, as described previously. Osteoclasts were incubated with ActinGreenTM488 fluorescent ghost pen cyclic peptide (Invitrogen, R37110, two drops into 1.5 ml PBS) for 30 min in dark at room temperature. After PBS washing, nuclei were stained for 5 min with DAPI (1:1000 dilution). F-actin rings were observed using an immunofluorescence microscope (ZEISS).

### ELISA

The levels of OPG, RANKL, IL-6, and TNF-α were evaluated in the culture medium supernatant of each group after 48 h of intervention in MLO-Y4 cells under varied circumstances using ELISA kits (R&D Systems). The O.D. was recorded at 450 nm using a fully automatic micro-enzymatic standard.

### Protein expression and western blotting analysis

After being treated with lysis buffer, osteocytes and osteoclasts were rinsed twice with PBS, centrifuged at 15,000 rpm for 15 min, and kept on ice for 20 min. After collecting the supernatant, the protein content was determined using the BCA Protein Assay Kit (Beyotime, P0010). Then, separated 50 μg of each sample by SDS-PAGE (10%) and electroblotted on PVDF membranes (membrane soaking in methanol for 3 min before protein transfer). The membranes were incubated with primary antibodies for sclerostin (Abcam, ab86465, 1:1000 dilution), β-catenin (dephosphorylated form, CST, 8480 T, 1:1000 dilution), OPG (Abcam, ab183910, 1:2000 dilution), RANKL (Abcam, ab45039, 1:1000 dilution), TNF-α (Proteintech, 17590–1-AP, 1:1000 dilution), IL-6 (Abcam, ab9324, 1:2000 dilution), NFATc1 (CST, 5861S, 1:2000 dilution), RANK (Abcam, ab200369, 1:1000 dilution), CTSK (Abcam, ab19027, 1:2000 dilution), and TRAP (Abcam, ab19146, 1:1000 dilution) overnight at 4 °C after being blocked with 5% BSA for an hour at room temperature. The membrane was washed with TBST (tris-buffered saline containing tween), followed by treatment with horseradish peroxide (HRP), goat anti-rabbit IgG, and goat anti-Rat IgG (Multisciences, GRT007, 1:2000 dilution) for 60 min at room temperature. The membranes underwent three additional TBST washes before being exposed to electrochemiluminescence (ECL) and subjected to GIS analysis. In addition, to evaluate the expression of the target protein, we chose β-actin as the internal reference protein.

### TRAP staining

Sections of skull specimens were prepared, as described above, and TRAP staining was performed on these sections, using the TRAP staining kit. In vitro, BMMs were inoculated at a concentration of 3 × 10^5^/well on 6-well plates, and then groups of BMMs were induced to differentiate toward osteoclasts, as described previously. TRAP staining and microscopy were performed to visualize the differentiation of these cells. Using Image Pro-Plus 6.0, TRAP-positive cells with 3 + nuclei were identified as osteoclasts, followed by counting them.

### Osteoclastic resorption assessment

The function of osteoclasts was assessed by the resorption assay. Briefly, BMMs were inoculated in osteo assay surface plates containing 24-wells (Corning, USA) at a concentration of 10 × 10^4^/well. Then groups of BMMs were induced to differentiate toward osteoclasts as before. After 6 days, trypsin digestion and three PBS washes were performed on the cells. Following that, images were captured with a simple light microscope (Zeiss), and Image Pro-Plus (Version-6.0) was employed to analyze the region of the resorption depression.

### Statistical evaluation

For each assay, three separate replicate experiments were conducted. The ANOVA and post hoc multiple comparisons (Tukey Kramer’s post hoc test) were used to identify differences between groups. The data have been presented as mean standard deviation (SD). SPSS version 27.0 was employed to analyze all the data, differences with *P* < 0.05 were considered significant.

## Results

### Inhibition of SOST gene attenuated Ti particles-induced skull osteolysis in mice

**Fig. 1:**
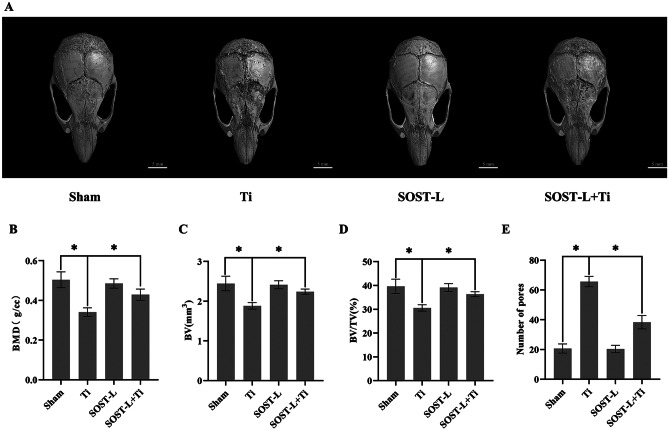
In vivo bone resorption caused by Ti particles are reduced at low levels of SOST. **A** A general perspective of each group’s 3D representation of the calvaria. Bone mineral density (BMD), bone volume (BV), bone volume as a proportion of total tissue volume (BV/TV), and the number of pores in each group’s quantification have been shown in **B**–**E**, respectively. Scale bar = 5 mm. * *P* < 0. 05

Micro-CT scan image analysis and 3D reconstruction showed that compared with the sham group, the Ti group showed increased bone erosion, significantly lower BV, BMD, BV/TV, an elevated quantity of osteolysis craters, and statistically (*P* < 0.05) difference was recorded. The SOST-L + Ti group showed decreased bone erosion, higher BMD, BV, and BV/TV, and a lower number of osteolysis craters, compared with the Ti group. Moreover, the difference was found to be statistically (*P* < 0.05). It indicated that inhibition of the SOST gene alleviated Ti particle-induced osteolysis (Fig. 1A–E).

**Fig. 2:**
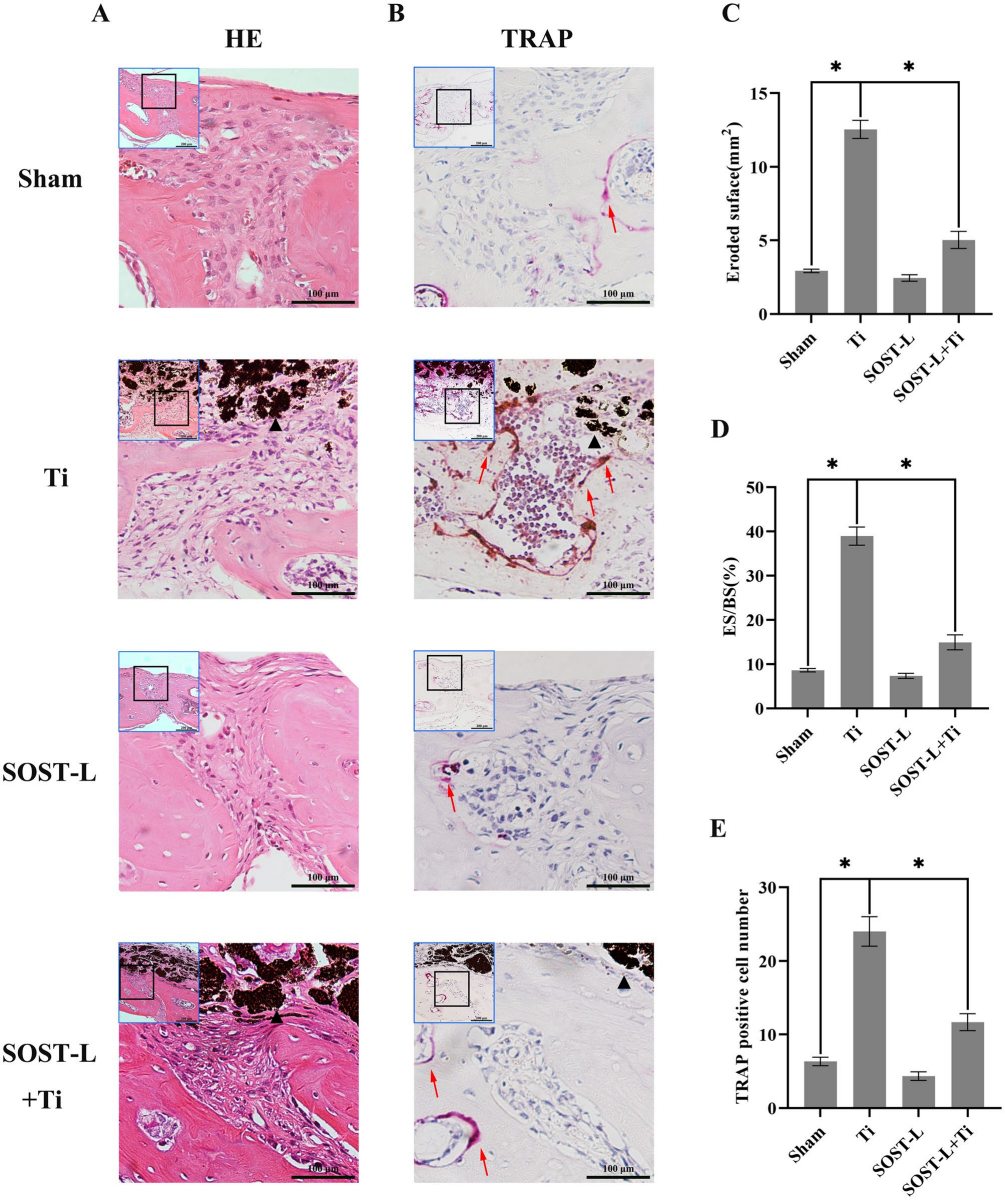
Histological staining and histomorphometric evaluation of SOST reduction on murine calvarial osteolysis. Herein, **A** and **B** represent H&E- and tartrate resistant acid phosphatase (TRAP)-stained calvarial slices, accordingly. **C** Evaluation of the eroded surface area histomorphometrically; **D** percentage of eroded surface per bone surface (ES/BS, %); **E** number of TRAP-positive osteoclasts (purple, indicated by red arrows). The black color triangle represents Ti particles. The thumbnail image on top left is 20 × magnified, scale bar = 200 µm, and the main image is 40 × magnified, scale bar = 100 µm. * *P* < 0.05

H&E staining bone morphometry showed that Ti particles led to more bone erosion area (ES) and a higher ES/BS ratio on the skull, compared to the sham group. While inhibition of the SOST gene decreased ES and the ratio of ES/BS, with statistical differences (*P* < 0.05), as depicted in Fig. 2A–D. TRAP stain showed that the positive osteoclasts with TRAP staining were considerably prominent under the stimulation of Ti particles. After SOST gene inhibition, the quantity of TRAP staining positive osteoclasts was decreased (Fig. 2B, E). Moreover, in the mouse skull lysis model, inhibition of the SOST suppressed osteoclast activation and attenuated Ti particle-induced osteolysis.

### SOST gene silencing improved β-catenin expression and decreased RANKL/OPG ratio in mouse cerebral osteolysis model

**Fig. 3:**
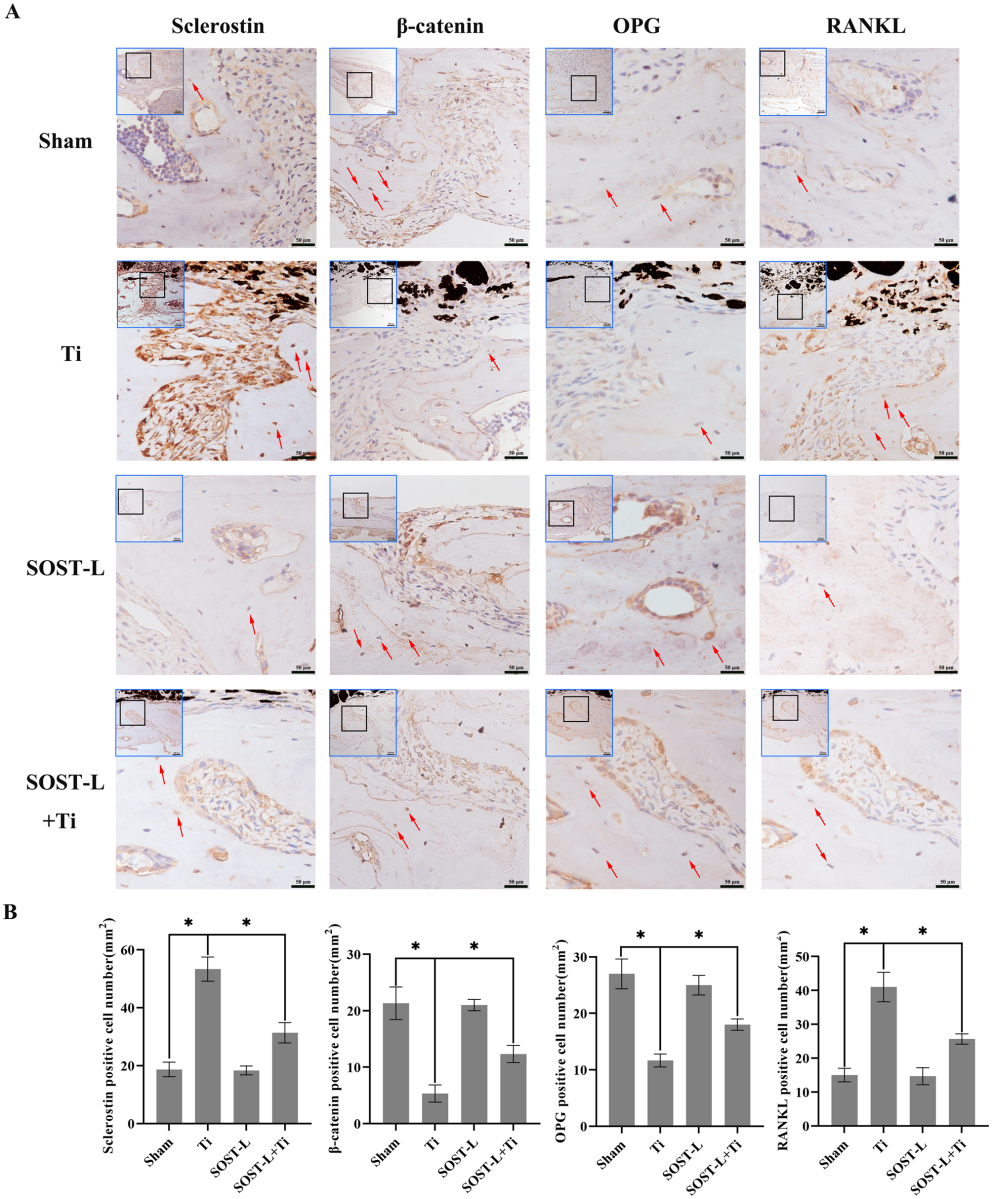
SOST reduction promotes the expression of β-catenin and OPG and inhibits the expression of RANKL in vivo. **A**–**B** Image Pro Plus 6.0 was used to evaluate sclerostin, β-catenin, OPG, and RANKL immunohistochemical staining in vivo (brown, indicated by red arrows). The thumbnail image on top left is 20 × magnified, scale bar = 100 µm, and the main image is 40 × magnified, scale bar = 50 µm. * *P* < 0.05

**Fig. 4:**
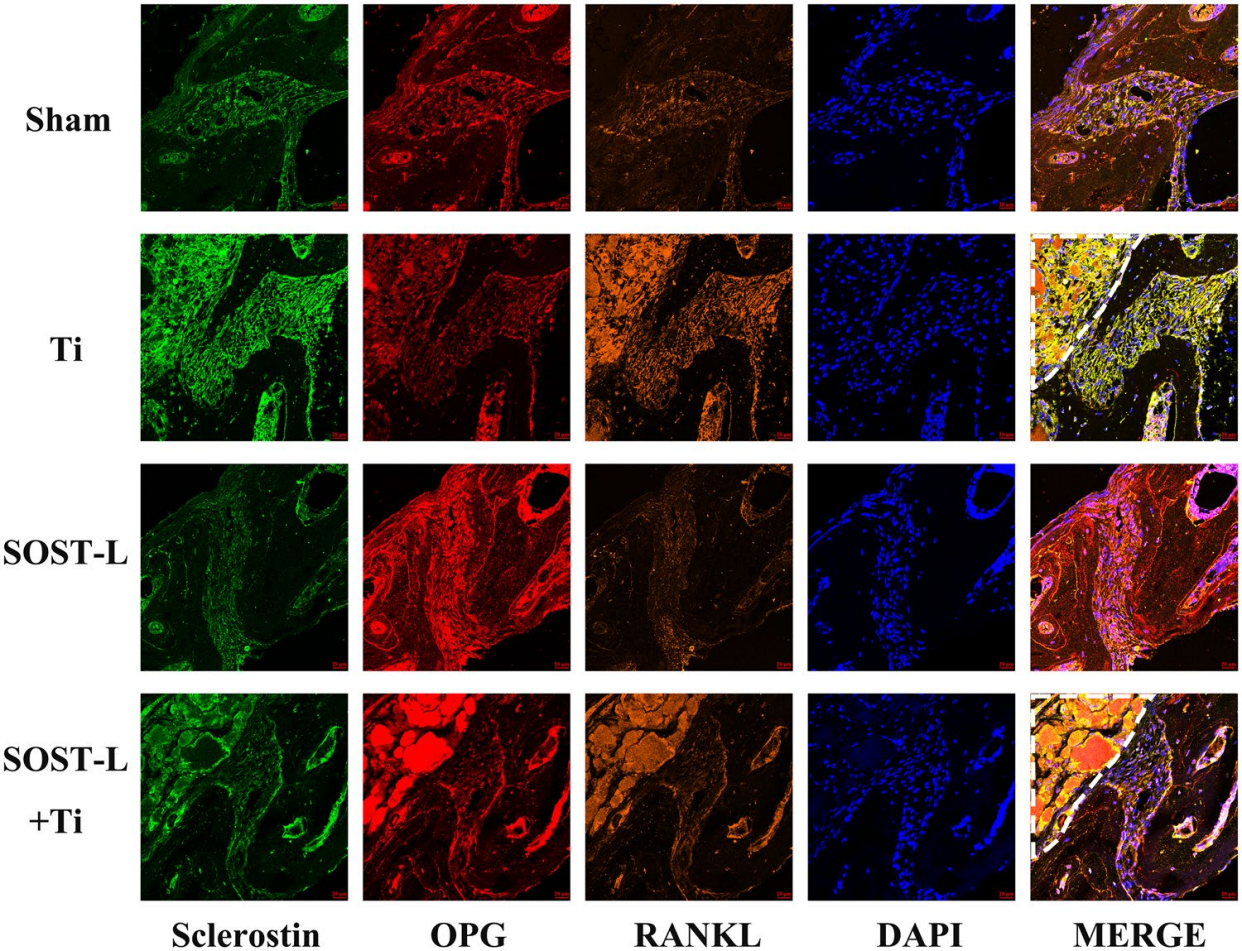
SOST reduction promotes the OPG expression level and inhibits the RANKL expression in vivo. Representative images of immunofluorescence staining(20 ×). Sclerostin (green), OPG (red), RANKL (orange), and nuclei (blue). Ti particles were showed in the white dashed line area, scale bar = 20 µm

The expression of β-catenin, osteoprotegerin (OPG), and RANKL in cranial bone was studied in depth to determine its role in osteolysis. IHC staining (Fig. 3A–B) and immunofluorescence (Fig. 4) assays showed that which has been compared to the sham group;Ti particle action increased sclerostin expression and RANKL in mouse cranial bone, and the β-catenin expression and OPG expression were considerably reduced. Sclerostin expression and RANKL levels were reduced by SOST gene inhibition, whereas β-catenin expression and OPG levels were increased with the same treatment with Ti particles (*P* < 0.05). These results demonstrated that in a mouse cranial osteolysis model, β-catenin expression was elevated and the RANKL/OPG ratio was lowered when the SOST gene was inhibited.

### The effect of increasing Ti particle concentration on sclerotin, β-catenin, TNF-α, IL-6, and the RANKL/OPG ratio in MLO-Y4 cells cultured in vitro

**Fig. 5:**
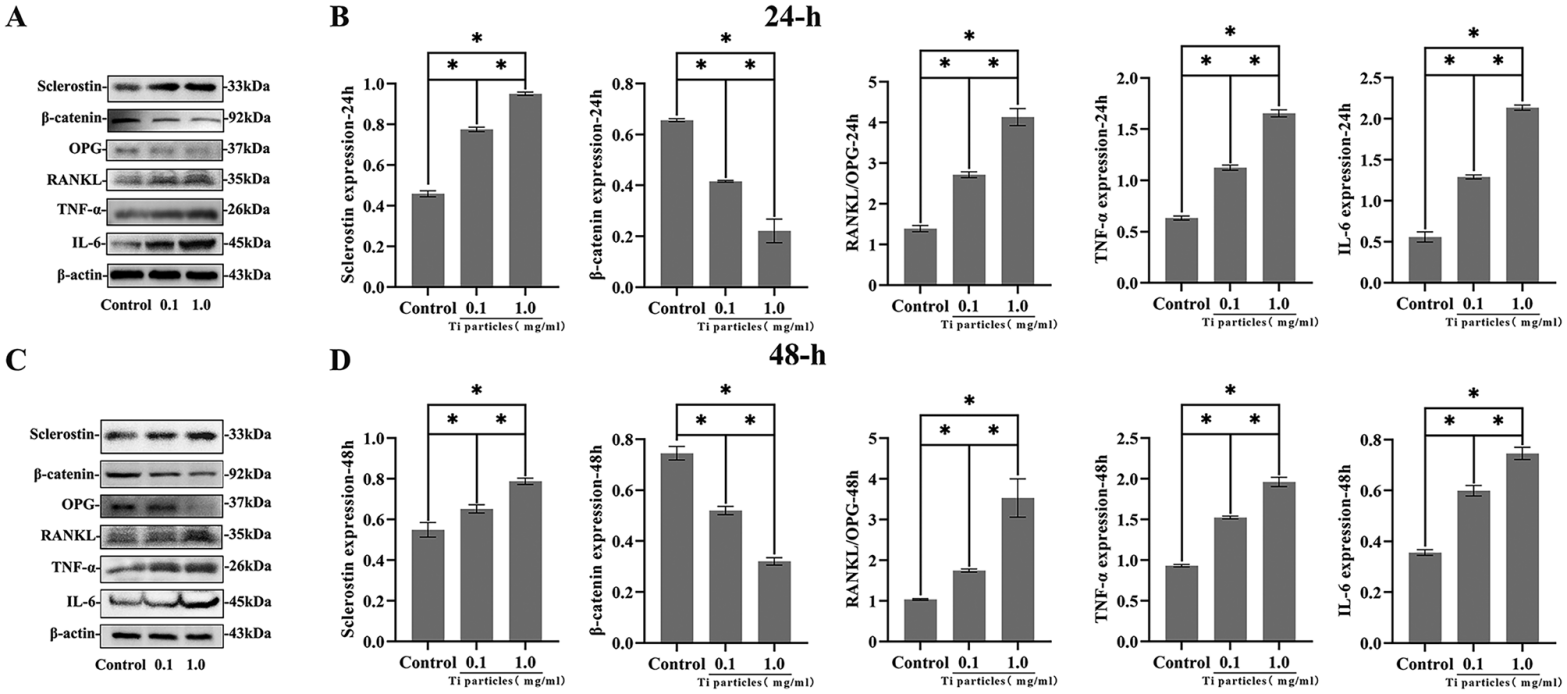
The impact of Ti particles on MLO-Y4 cells. **A**–**D** The proteins, including sclerostin, β-catenin, RANKL/OPG, TNF-α, and IL-6 expression levels in MLO-Y4 cells exposed to various Ti particle concentrations for 24 and 48 h. Western blotting was employed to quantify sclerostin, β-catenin, and the ratio of RANKL/OPG, TNF-α, and IL-6. Here, β-actin was used as a standard protein. * *P* < 0.05

In this study, MLO-Y4 cells were exposed to Ti particles at varying concentrations for 24 and 48 h, respectively. After exposing MLO-Y4 to Ti particles, the expression of sclerostin was found to be elevated, whereas the expression of β-catenin was found to be progressively lowered as the concentration of Ti particles was raised. Simultaneously, the ratio of RANKL/OPG also increased, and the differences were statistically considerable (*P* < 0.05, Fig. 5A–D). The changes in sclerostin and β-catenin expression in vitro assays for exposure of MLO-Y4 cells to Ti particle were consistent with those in the particle-induced mouse skull osteolysis model. In addition to these changes, with rising Ti particle concentrations, TNF-α, and IL-6 expression levels also increased (*P* < 0.05, Fig. 5A–D). Inflammatory mediators including TNF-α, and IL-6 are upregulated by Ti particles, which may have an effect on wear particle-induced osteoclast-genesis and lead to increased osteolysis.

### SOST Inhibition reduced the expression of RANKL/OPG, TNF-α, and IL-6 in the medium and attenuated the effect of Ti particles on MLO-Y4 osteocytes. Whereas after SOST overexpression, similar changes to those after Ti particle treatment occurred

In this study, the SOST gene of MLO-Y4 was suppressed or overexpressed using lentiviral transfection. Furthermore, western blotting was employed to confirm the outcomes of in vivo experiments (Supplement 1A). Additionally, the most potent sequences of ShRNA-2 and SOST-H were selected for the in vitro cellular experiments.

**Fig. 6:**
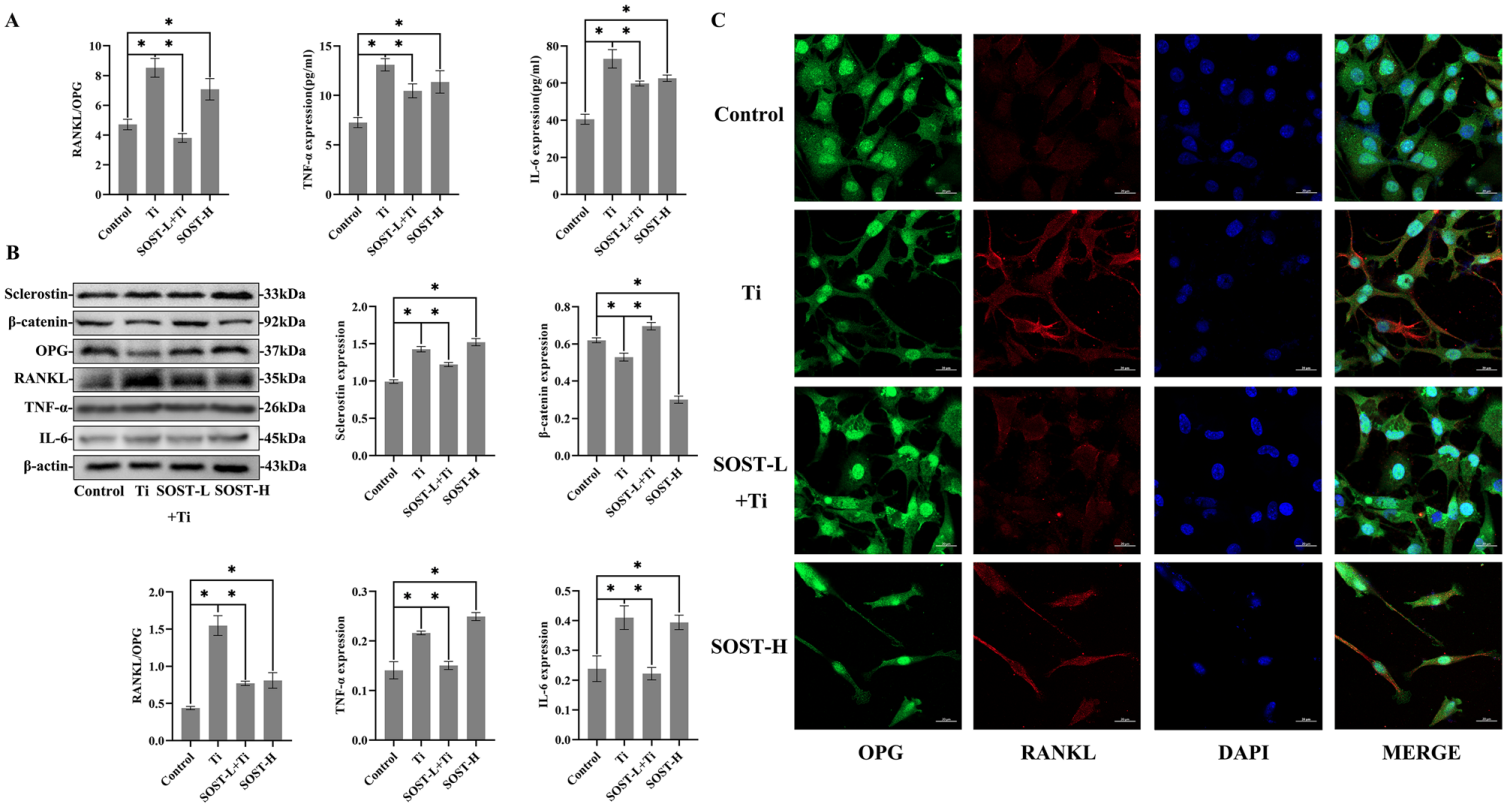
Ti particles elevate the expression levels of sclerostin, the ratio of RANKL/OPG, TNF–α, and IL-6 but decreased the β-catenin expression level. However, SOST reduction inhibited these effects. **A** The ratio of RANKL/OPG, TNF-α, and IL-6 protein concentrations in the culture medium of different MLO-Y4 groups was identified by ELISA. **B** Sclerostin, β-catenin, and the ratio of RANKL/OPG, TNF-α, and IL-6 concentrations were evaluated by western blotting. And β-actin was used as a standard protein. **C** OPG and RANKL expression in osteocytes by immunofluorescence analysis at 48 h. OPG (green), RANKL (red), and nuclei (blue). Scale bar = 20 µm, * *P* < 0.05

According to ELISA results, the levels of RANKL/OPG, TNF-α, and IL-6 were higher in MLO-Y4 osteocytes tested through Ti particles for 48 h, compared to the control group (*P* < 0.05). Afterward, SOST inhibition, the levels of RANKL/OPG, TNF-α, and IL-6 were decreased even under Ti particle intervention (*P* < 0.05). Meanwhile, when SOST was overexpressed, the levels of RANKL/OPG, TNF-α, and IL-6 in MLO-Y4 osteocytes were increased, showing similar changes in MLO-Y4 osteocytes treated with Ti particles (Fig. 6A).

It was revealed by western blotting that after SOST inhibition, the SOST-L + Ti group had a significantly lower RANKL/OPG ratio and higher expression of β-catenin, compared to the Ti group. TNF-α and IL-6 expression was also downregulated (*P* < 0.05). Although β-catenin expression was reduced in MLO-Y4 osteocytes following SOST overexpression, the RANKL/OPG ratio, TNF-α, and IL-6 levels were all considerably increased (*P* < 0.05, Fig. 6B).

Due to staining with immunofluorescence, similar consequences were shown: OPG expression (in green color) decreased, and RANKL expression, shown in red, increased with Ti particle stimulation or SOST overexpression. However, OPG expression increased and RANKL expression decreased after SOST inhibition (Fig. 6C).

### Ti particles induced in MLO-Y4 cells a differentiation of osteoclasts and an induced bone resorption. Low expression of SOST in MLO-Y4 inhibited the differentiation of osteoclasts and resorption of bones induced by Ti particle stimulation, whereas SOST overexpression produced similar changes to Ti particle stimulation

To further investigate the effects of SOST changes in MLO-Y4 induced via Ti particle on osteoclasts, different culture solutions of MLO-Y4 cells were used to induce differentiation of BMMs toward osteoclasts as a way to mimic the regulation of osteocytes on osteoclasts.

**Fig. 7:**
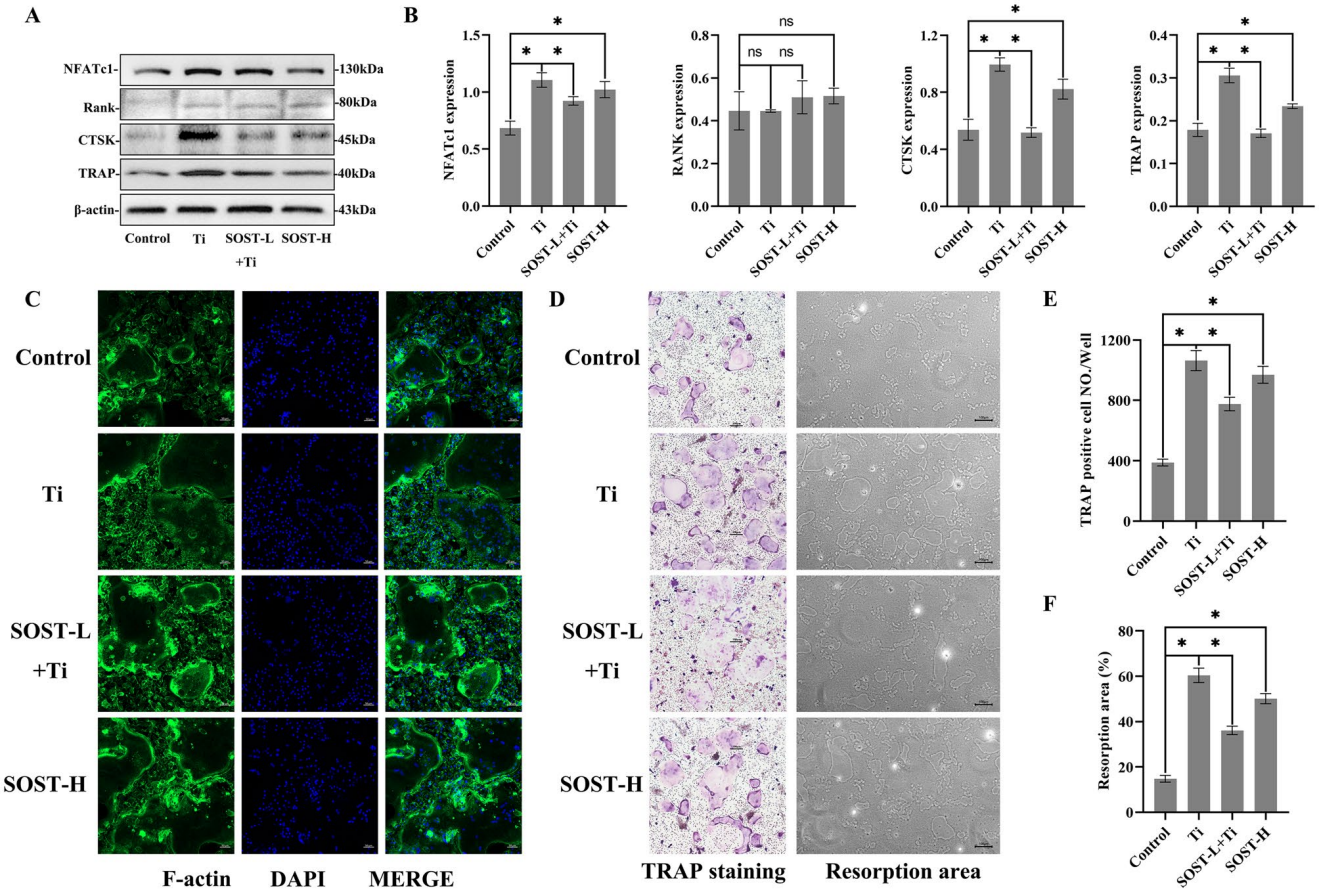
Effects of the culture medium of MLO-Y4, which was treated under different conditions, on BMM’s differentiation into osteoclasts. **A**–**B** Western blot detection of NFATc1, RANK, CTSK, and TRAP protein levels normalized to β-actin. **C** Multinucleated cells stained with F-actin were visualized by immunofluorescent staining. **D** BMMs induced by different culture media stained with TRAP and resorption areas; **E** TRAP-positive positive cell number per well (≥ 3 nuclei). **F** Area of resorptive pits. * *P* < 0.05. ^ns^ Non-significant

In a western blotting analysis, the Ti group outperformed the control group in terms of osteoclast protein expression of NFATc1, CTSK, and TRAP (*P* < 0.05). However, the SOST-L + Ti group reduced the protein expression of NFATc1, CTSK, and TRAP when compared to the Ti group (*P* < 0.05). In the SOST-H group, MLO-Y4 osteocytes with SOST overexpression, the expression of NFATc1, CTSK, and TRAP increased, which showed similar changes to the Ti group (Fig. 7A–B). The expression of RANK in each group had no significant differences.

According to the fluorescent staining, induction of BMMs from the culture solution of MLO-Y4 osteocytes exposed to Ti particles stimulated the synthesis of F-actin rings in mature osteoclasts (shown in green). However, SOST inhibition reduced the formation and structure of such rings (Fig. 7C).

When compared to the control group, the Ti group had more positive osteoclast TRAP-stained cells than the control group (*P* < 0.05). In the SOST-L + Ti group, the number of TRAP-positive cells was lower than in the Ti group (*P* < 0.05), and in the SOST-H group, the number of TRAP-positive cells was significantly higher, showing similar changes to the Ti group (Fig. 7D, E). SOST inhibition of MLO-Y4 attenuated osteoclast differentiation induced through Ti particles.

The effects of bone resorption led to a high bone resorption area on tooth slices caused by osteoclasts in the Ti group relative to the control group (*P* < 0.05) as a result of bone resorption. When compared to the Ti group, the SOST-L + Ti group’s bone resorption area was considerably decreased (*P* < 0.05). The SOST-H group, which demonstrated a change similar to that in the Ti group, had a significantly increased bone resorption area (Fig. 7D–F).

## Discussion

Multiple studies have shown that aseptic loosening, along with periprosthetic osteolysis, is a major cause of arthroplasty failure and revision [1–7, 15]. In contrast, wear particles produced around the prosthesis are one of the most important factors in periprosthetic osteolysis. Osteolysis is the result of several reactions set off by wear particles surrounding the prosthesis that disturb the dynamic equilibrium between bone formation and bone resorption. Wear particles activate inflammatory cells around the prosthesis to release various inflammatory mediating substances, such as TNF-α and IL-6. They further activate osteoclasts, leading to increased bone resorption [18, 19]. Simultaneously, inflammatory mediators also inhibit osteoblast function, leading to decreased bone formation [20, 21]. The generation of wear particles on and around the prosthetic surface cannot be eliminated, despite ongoing advancements in prosthesis design options and materials [3, 22]. In view of this fact, inhibiting periprosthetic bone resorption and/or increasing bone formation is the main starting point for the current treatment of osteolysis due to wear particles.

Osteocytes are the main class of bone cells and are terminally differentiated osteoblasts that are deeply embedded in the bone matrix. Numerous studies have demonstrated that osteoclasts and osteoblasts can communicate with osteocytes via their dendritic and tubular structures, allowing osteocytes to participate in regulating bone formation and bone resorption [23, 24]. Because it controls bone formation and bone resorption [11], the production of SOST/sclerostin in bone, which is markedly carried out by osteocytes, has gained particular attention. Meanwhile, osteocytes are essential sources of the RANKL, and osteocyte RANKL is responsible for the bone loss associated with unloading [10, 25]. RANKL and IL-6 promoted osteocytes to induce osteoclast differentiation [26, 27]. The regulation of osteocytes on osteoclast and osteoblast function during bone remodeling becomes a new development in the periprosthetic osteolysis procedure. According to our reported study, inhibition of SOST gene expression of osteocyte activated the Wnt/β-catenin signaling pathway and promoted differentiation of osteoblast, thereby promoting bone formation and attenuating bone loss caused by Ti particles [15, 16]. However, the effects of osteocyte and sclerostin on periprosthetic osteolysis remain elusive. In this view, the effect of SOST on osteoclast function and bone resorption has been investigated further and the regulation of osteocytes on osteoclast has also been regulated.

Herein, the results obtained from in vivo study confirmed that Ti particles enhanced sclerostin levels and the ratio of RANKL/OPG, and enhanced the cell quantity of positive TRAP staining. In distinction, osteolysis was reduced, the β-catenin level increased, RANKL/OPG reduced, and the number of positive TRAP cells declined after SOST inhibition. These findings suggested that SOST inhibited Wnt signaling pathway and activated osteoclast function to lead to osteolysis. Furthermore, SOST reduction protected against osteolysis caused by wear particles by inhibiting bone resorption. It has been shown that osteocytes can regulate osteoclasts via the Wnt/ß-catenin signaling pathway.

To further validate the abovementioned results, the interactions between Ti particles and osteocytic MLO-Y4 cells were evaluated in vitro. Because of osteocyte exposure, Sclerostin levels rose in the presence of Ti particles, decreased β-catenin expression, elevated RANKL/OPG ratio, and the expression of TNF-α and IL-6 in vitro. By contrast, low expression of SOST resulted in increased β-catenin expression, lower RANKL/OPG ratio, and decreased the level of TNF-α and IL-6. OPG/RANKL/RANK system acts as a key role in osteoclast differentiation and formation through MAPK and NF-kB cascades [28–30]. A significant source of RANKL produced by osteoclasts is osteocytes. Two comprehensive independent studies [10, 31] have shown that selective deletion of the RANKL gene Tnfsf11 in engineered mouse osteocyte populations (but not osteoblasts or precursors) results in a defective osteoclastogenic phenotype in global mutants. OPG, a soluble RANK decoy receptor that inhibits the formation of osteoclasts, is a component that is primarily produced by osteocytes [32]. Thus, osteocytes positively control osteoclastogenesis by increasing RANKL expression and decreasing OPG expression, or conversely, can reverse the ratio to decrease resorption activity. Likewise, mediate inflammation such as TNF-α and IL-6 have a negative impact on periprosthetic osteolysis by activating osteoclastic differentiation and function [33–35]. Therefore, decreased release of the inflammatory mediators and reduced ratio of RANKL/OPG can also overcome bone resorption.

The indirect co-culture model was created using a medium from osteocyte culture-treated bone marrow-derived monocyte-macrophages to see if the Ti particles’ induced change in SOST expression of osteocytes was followed by a corresponding change in their RANKL/OPG, TNF-α, and IL-6 levels, and whether this affected the function of osteoclasts. The level of osteoclast-related proteins, including NFATc1, RANK, CTSK, TRAP, and F-actin, and the number of positive TRAP cells and the bone resorption area have considerably increased in the Ti-treated osteocyte group. These manifestations of osteoclast were inhibited after SOST reduction, compared to the Ti-treated osteocyte group. However, SOST overexpression promoted osteoclast differentiation and maturation. The results indicated that SOST might serve a considerable role in regulating bone resorption and osteoclast differentiation in process of periprosthetic osteolysis.

Some studies showed that the Wnt/β-catenin signaling pathway participates in controlling the expression of RANKL and OPG [36, 37]. And sclerotin, which is a natural inhibitor of the canonical Wnt pathway, induced bone resorption by osteoclasts through the change in the ratio of RANKL/OPG [17, 38]. SOST was downregulated for the osteogenic response to mechanical loading and SOST overexpression reduced bone mass and strength by slowing down bone formation [39]. Virdi et al. found that treatment with sclerostin antibodies resulted in improved implant fixation in the osteoporosis model [40]. Due to its enhanced anabolic effect on bone formation and its diminished catabolic effect on bone resorption, sclerostin antibody therapy has been proposed as a new treatment for postmenopausal osteoporosis [41, 42]. Our previous [15, 16] and present study demonstrated that inhibition of SOST stimulated bone formation and impeded bone resorption through the model of particle-induced osteolysis in vivo and in vitro, which was similar to the results of Liu et al. [43]. In addition, our study also found inhibition of SOST expression decreased TNF-α and IL-6 levels, which reports were rare. According to Marahleh et al., TNF-a directly increases osteocyte RANKL expression and encourages the formation of osteoclasts [44]. Similar to this, Wu et al. showed that IL-6 increased in vitro RANKL activity osteocyte-mediated osteoclastogenesis [45]. In other words, decreased SOST expression also indirectly reduced osteocyte RANKL/OPG ratio and inhibited osteoclast formation by falling the level of TNF-α and IL-6. There is also a report that the IL-6 family produced by osteoclasts acts on osteocytes to reduce the expression of sclerostin [46, 47]. Therefore, there may be other interaction mechanisms between IL-6 and SOST, which are worthy of further study. Overall, our study demonstrates that inhibition of osteocytic SOST expression inhibits osteoclast formation, thereby depressing bone resorption and attenuating wear particle-induced osteolysis. The dual effects of suppression osteocytic SOST promoting bone formation and inhibiting bone resorption might make sclerostin antibody to be a new treatment against periprosthetic osteolysis.

However, our study has a few limitations. Although the mouse cranial osteolysis model has been considered a well-established model of osteolysis [48, 49], it differs from surgery of joint replacement in that the models were unable to fully simulate the environment in which the prosthesis is exposed to fluid pressure and mechanical loading. And future studies will further explore how osteoclasts affect osteocytes through feedback mechanisms.

## Conclusion

Taken together, this study showed that reduced SOST of osteocytes can inhibit the development of osteoclast by triggering the Wnt/β-catenin signaling cascade as well as suppressing RANKL/OPG ratio and the expression of inflammatory mediators, consequently minimizing the bone loss caused by wear particles.

## Supplementary Information

Below is the link to the electronic supplementary material.

Supplementary file2 (DOCX 95441 KB)

## Data Availability

The authors confirm that the data supporting the findings of this study are available within the article.

## Availability of data and materials

All the data generated or analyzed during this study are included in this article. The datasets used and/or analyzed during the current study are available from the corresponding author on reasonable request.

## Abbreviations

TRAP: Tartrate‑resistant acid phosphatase
Wnt: Wingless/integrated
Ti: Titanium
RANKL: Receptor activator of nuclear factor-κB ligand
OPG: Osteoprotegerin
RANK: Receptor activator of nuclear factor-κB
TNF-α: Tumor necrosis factor-α
IL-6: Interleukin-6
MMP-2: Matrix metallopeptidase-2
PBS: Phosphate buffered saline
C57/6: C57 black 6
CT: Computed tomography
ROI: Region of interest
BV: Bone volume
BMD: Bone mineral density
BV/TV: Bone volume/tissue volume
EDTA: Ethylenediaminetetraacetic acid
H&E: Hematoxylin and eosin
BS: Surface area of bone tissue
ES: Eroded bone surface area
MLOY4: Murine long bone osteocyte-Y4
α-MEM: Minimum essential medium alpha modification
FBS: Fetal bovine serum
shRNA: Short hairpin RNA
BMMs: Bone marrow-derived monocyte macrophages
M-CSF: Macrophage colony stimulating factor-1
PBST: Phosphate buffered saline containing tween
BSA: Bovine serum albumin
DAPI: 4',6-Diamidino-2-phenylindole
F-actin: Fibros actin
ELISA: Enzyme linked immunosorbent assay
SDS-PAGE: Sodium dodecyl sulfate poly acrylamide gel electrophoresis
PVDF: Polyvinylidene fluoride
NFATc1: Nuclear factor of activated T cells-1
CTSK: Recombinant cathepsin K
TBST: Tris-buffered saline containing tween
HRP: Horseradish peroxide
IgG: Immunoglobulin G
ECL: Electrochemiluminescence
