# Supplementary material for: SOST gene suppression stimulates osteocyte Wnt/β-catenin signaling to prevent bone resorption and attenuates particle-induced osteolysis
Source: J Mol Med (Berl). 2023 May 1;101(5):607–20. doi: 10.1007/s00109-023-02319-2 (PMC10163143; doi:10.1007/s00109-023-02319-2)
Supplement: Supplementary file 1 — Supplementary file2 (DOCX 95441 KB) [file 109_2023_2319_MOESM1_ESM.docx]

**Additional file: Supplement figure 1. Validation, screening, knockdown, and overexpression of SOST. Validation of the feasibility of the osteoclast induction method.** (A) The effectiveness of SOST-shRNA was determined following lentiviral transfection by assessing the expression of the sclerostin protein using western blotting. When compared to the scramble group, sclerostin in the shRNA-2 group was found to be significantly lower and increased in the SOST-H group. Moreover, for SOST transfection studies, the second and fourth shRNA sequences, i.e., shRNA-2 and SOST-H, were used. (B) Detection of osteoclast-specific protein expression at different periods. (C) Multinucleated cells stained with F‐actin and visualized immunofluorescent staining. *P < 0.05.

**Additional file: Supplement figure 2. SOST reduction promotes the OPG expression level and inhibits the RANKL expression in vivo.** Representative images of immunofluorescence staining(10×). Sclerostin (green), OPG (red), RANKL (orange), and nuclei (blue). Ti particles were showed in the white dashed line area, scale bar = 50 µm.


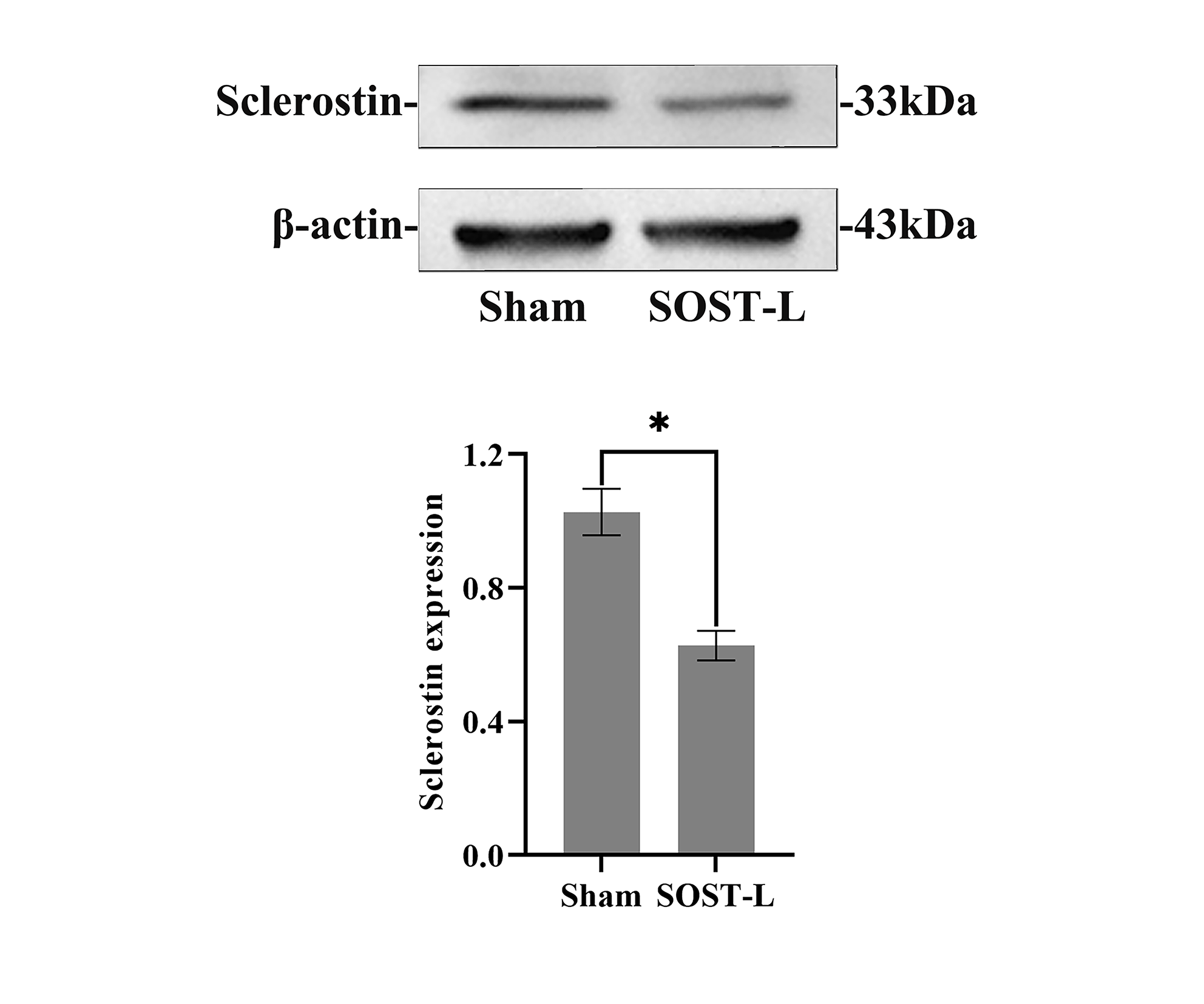


**Additional file: Supplement figure 3. Validation of the efficiency of viral vector infection in vivo experiments.** The effectiveness of SOST-shRNA was determined following adeno-associated virus transfection by assessing the expression of the sclerostin protein using western blotting. When compared to the Sham group, sclerostin in the SOST-L group was found to be significantly reduced. β-actin was used as a standard protein. *P < 0.05.

**Additional file: Supplement table 1. SOST-shRNA and SOST overexpression sequences information**

| Types of virus: | lentivirus |
| --- | --- |
| Type of vector: | LV8N(EF-1aF/mCherry&Puro) |
| SOST-shRNA sequence (5'-3') | GACAGCATATCTTACATTAAA |
| SOST overexpression sequence (5'-3'): | https://www.ncbi.nlm.nih.gov/nuccore/NM_024449.6  atgcagcc ctcactagcc  061 ccgtgcctca tctgcctact tgtgcacgct gccttctgtg ctgtggaggg ccaggggtgg  121 caagccttca ggaatgatgc cacagaggtc atcccagggc ttggagagta ccccgagcct  181 cctcctgaga acaaccagac catgaaccgg gcggagaatg gaggcagacc tccccaccat  241 ccctatgacg ccaaagatgt gtccgagtac agctgccgcg agctgcacta cacccgcttc  301 ctgacagacg gcccatgccg cagcgccaag ccggtcaccg agttggtgtg ctccggccag  361 tgcggccccg cgcggctgct gcccaacgcc atcgggcgcg tgaagtggtg gcgcccgaac  421 ggaccggatt tccgctgcat cccggatcgc taccgcgcgc agcgggtgca gctgctgtgc  481 cccgggggcg cggcgccgcg ctcgcgcaag gtgcgtctgg tggcctcgtg caagtgcaag  541 cgcctcaccc gcttccacaa ccagtcggag ctcaaggact tcgggccgga gaccgcgcgg  601 ccgcagaagg gtcgcaagcc gcggcccggc gcccggggag ccaaagccaa ccaggcggag  661 ctggagaacg cctactag |
